# Comparing imaging modalities and healing criteria for the estimation of fracture age: a systematic review

**DOI:** 10.1007/s00414-025-03589-w

**Published:** 2025-10-06

**Authors:** Charlotte G. Lawrence, Mark A. Williams, Charlotte Primeau

**Affiliations:** Forensic Centre for Digital Scanning and 3D Printing, WMG, University of Warwick, Coventry, CV4 7AL UK

**Keywords:** Fractures, Healing, Physical abuse, Medical imaging, Digital imaging, Time since injury, Timeline, Forensics, Ageing

## Abstract

The estimation of fracture age is important for both clinical and forensic purposes. The objective of this systematic review is to identify the imaging modalities currently used in clinical and forensic practice for fracture ageing, with a view to appraise technologies used, patient demographics studied in research, and the healing stages and criteria defined to estimate time since injury. After conducting a bibliographic literature search, we identified 21 suitable publications for inclusion in the review. Comparison of the literature for fracture ageing found that most research has used 2D radiography, predominantly using an antemortem paediatric population. The remaining publications used MRI, CT, histology, and macroscopy. Although the most frequent number of radiographic stages assessed was six, no two methods assessed the same combination of features. Indeed, variation in the number and definition of healing stages rendered comparison between the publications challenging. Consequently, limited work has been carried out to validate existing methods of ageing. The results therefore reiterate the need for caution in the use of radiographic modalities for forensic fracture ageing. Histology remains the undisputed gold standard, however it is a destructive and exclusively postmortem method. As such, there is need for further research to investigate the potential of additional imaging modalities such as micro-CT, with a high-resolution, 3D, and non-destructive nature. This can serve as a valuable complement to help support and navigate the challenges associated with traditional histopathological methods.

## Introduction

Fractures are a common consequence of traumatic injuries, and their identification and analysis can yield information that is important for both clinical and forensic investigations. In a clinical environment, the diagnosis of fractures is essential to identify appropriate treatment pathways, and later monitor their effectiveness based on the progress of healing to consider potentially adjusting treatment [1]. Alternatively in forensic investigations, the detection of skeletal injuries can offer insights into the circumstances surrounding traumatic events and, when relevant, help establish cause of death. For both living and deceased individuals, this may be a single traumatic incident, or in cases of suspected abuse, can include repeated injuries sustained over an extended period [2]. Indeed, identifying a timeline over which injuries occurred based on extent or lack of healing can help clarify the persons involved; such as when a child or vulnerable adult spent time with different caregivers [3]. Such conclusions also yield important investigative implications for determining subsequent charges against the persons responsible (e.g., homicide, voluntary vs. involuntary manslaughter, infanticide, child cruelty, and neglect [4]).

Fracture ageing in both clinical and forensic contexts is extremely challenging. Although a collection of biologic features are associated with notable events in healing, the rate at which an individual progresses through these is variable, often with considerable overlap between phases [3, 5]. Biological variation such as age and health status are often cited to affect the temporality of healing [5–7], however the small volume of published data on each demographic group renders it difficult to quantify this effect. Severity and context of the fracture origin may also affect the progression of healing and therefore age estimation. This is a particular issue in cases of abuse where individuals may experience a delay in their access to medical treatment, allowing the fracture to remain mobile as the bone continues to be used [3, 5, 8]. This can result in malunion of the fracture fragments or delayed healing [3, 5], and cause a single injury to exhibit several stages of healing simultaneously [9].

The purpose of this systematic review is to compare the imaging modalities currently used to estimate the age of bone fractures, with a view to identify future directions in fracture analysis. It will build upon the previous reviews by Prosser et al. [3] and Messer, et al. [8], which focussed on the radiological dating specifically of paediatric fractures and expand this to consider the imaging modalities themselves and their use on patients of all ages. Thus, this review will clarify which imaging modalities are currently used for estimating fracture age, the patient demographics studied, as well as the healing stages and criteria used by practitioners to define time since injury. The review also aims to capture a holistic overview of the imaging modalities and analytic approaches currently used for fracture ageing and envisage which modalities may form the focus of future research.

## Materials and methods

### Search strategy

A literature search of six electronic databases was performed in summer 2024, screening the earliest available records up until July 2024. Databases searched were Scopus, ProQuest, Web of Science, PubMed, Ovid (encompassing Embase and Embase Classic), and EBSCOhost (covering EBSCOhost eBook collection, Child Development & Adolescent studies, CINAHL, and MEDLINE). The following search query was used: (bone or skele*) and (ag? ing or dating or time or interval) and (fracture or injury or trauma or callus) and (heal*) and (post*mortem or forensic). Asterisk (*) was used to enable truncated terms to be searched for words with multiple endings, such as heal* used in place of ‘heal’, ‘healed’ and ‘healing’. Question mark (?) was used to ensure words with variable spelling were included, with ag? ing used to retrieve results for both ‘aging’ and ‘ageing’.

### Paper selection and data extraction

Papers were selected based on the following inclusion criteria:

I. Be disseminated in a peer-reviewed English language publication,
II. Be based on data from human populations,
III. Have a focus on the methodology for ageing fractures (i.e., not a clinical or forensic case report),
IV. Offer clearly defined stages or criteria for ageing fractures,
V. Include a timeframe associated with fracture healing stages/criteria.

**Fig. 1:**
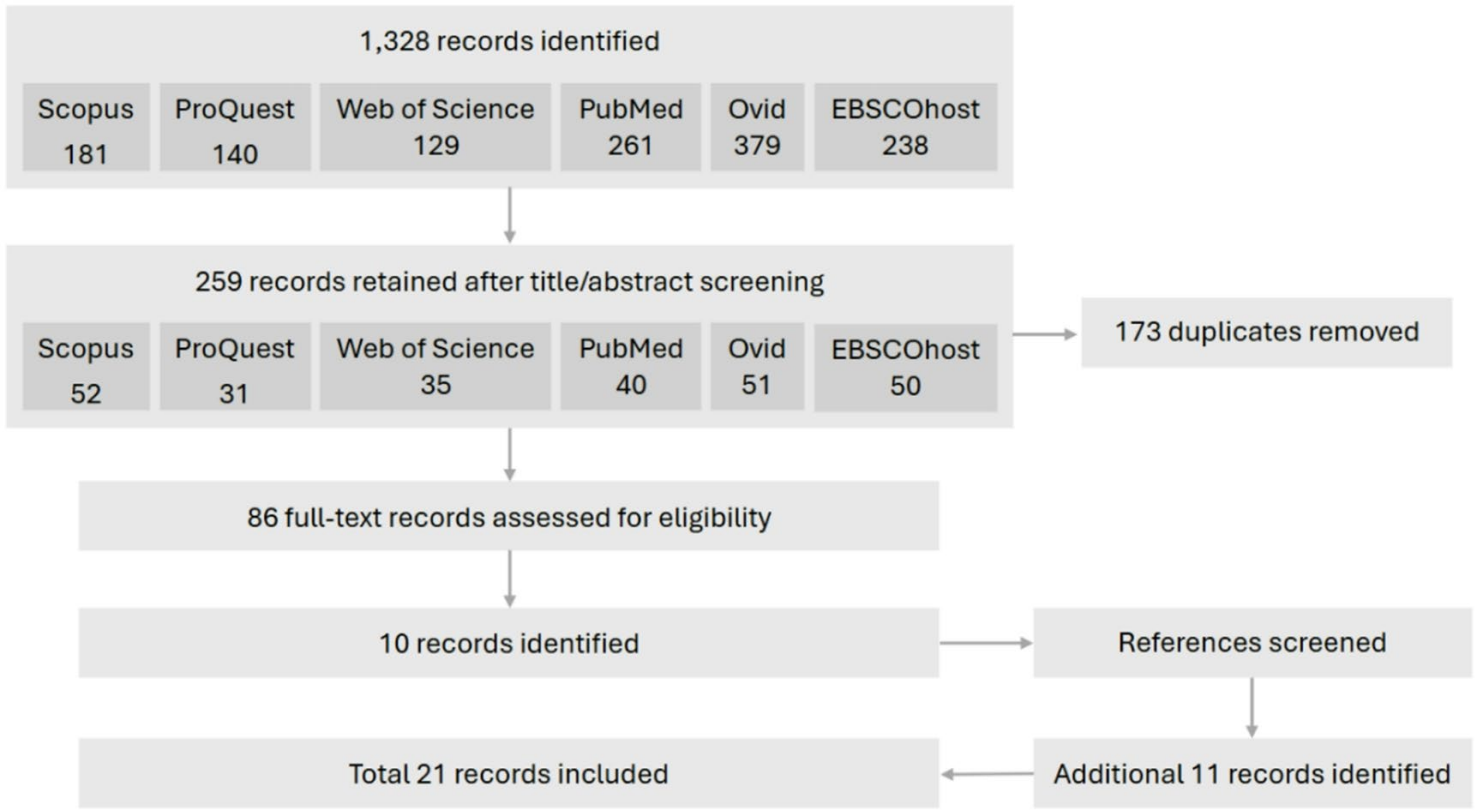
PRISMA flowchart of the publication selection method for this systematic review

The workflow, following the Preferred Reporting Items for Systematic Reviews and Meta-analyses guidelines (PRISMA) [10], can be seen in Fig. 1. A total of 1,328 results were returned after input of the search terms across all six databases. The title and abstract of each publication were then screened to assess eligibility according to inclusion criteria, yielding 259 potentially relevant papers, from which 173 duplicates were manually removed. The 86 remaining texts were assessed for eligibility, and if their inclusion was uncertain, they were reviewed by a second author to reach a final decision. 10 of the 86 papers satisfied the inclusion criteria. The references within these were then screened, yielding a further 11 publications, totalling a final sample of 21 publications. The papers were then assessed to compare the year of publication, demographic information about the study sample and method of fracture ageing; including imaging modalities and the criteria defined to assess healing.

## Results

### Context and demographics of the literature

**Fig. 2:**
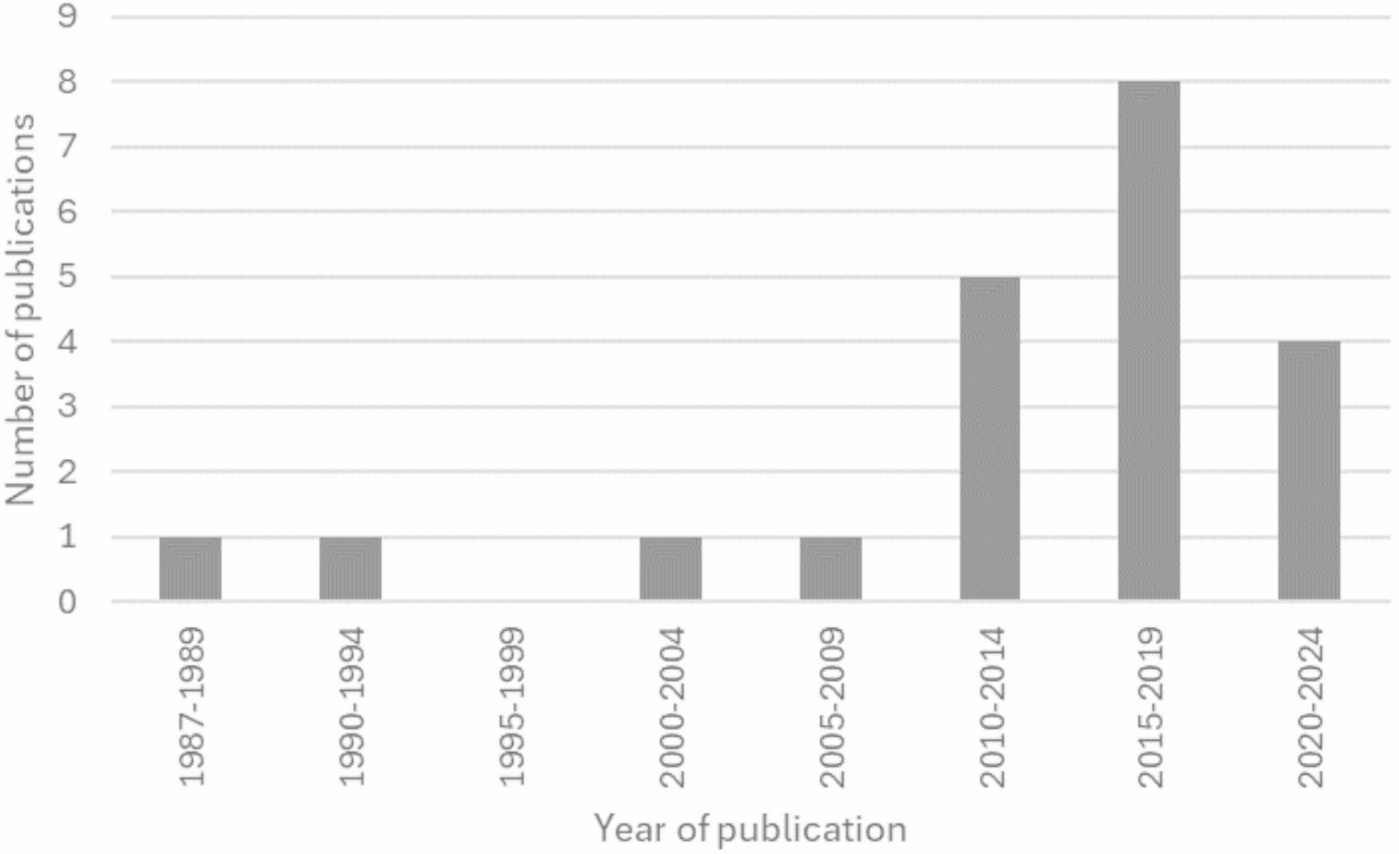
Bar chart illustrating the number of papers published each year (in five-year intervals) included in this systematic review

The 21 peer-reviewed publications covered 37 years of research from 1987 until 2024 (Fig. 2). For the first 23 years, between 1987 and 2010, only four works were published, starting with O’Connor & Cohen [11] and their book chapter ‘Dating fractures’ within Kleinman’s 1987 book ‘Diagnostic Imaging of Child Abuse’. Between 2011 and 2023, however, a steady increase is evident, with a further 16 papers published, averaging 1.23 papers per year.

Comparing the context and aims of the 21 publications, 19 papers [6, 12–29] studied a defined sample of fractures, while the remaining two book chapters [11, 30] drew on the existing literature and clinical experience to propose timetables for ageing. Of the 19 papers with a defined fracture population, 14 papers (73.7%) studied fractures in living patients [6, 13, 15, 17–21, 24–29], while five papers (26.3%) assessed fractures in postmortem cases [12, 14, 16, 22, 23]. Of the five postmortem samples, two were from archaeological human remains dating from the 17th to 20th centuries [12, 16], while three publications documented trauma from modern medicolegal samples [14, 22, 23].

Considering the age of the patients studied in each publication, 15 papers (71.43%) focused exclusively on a paediatric population, with ages ranging from birth until 18 years [6, 11, 15, 17–21, 23–25, 27–30]. A further 5 (23.81%) studied only adults, with ages ranging from 19 to 97 years [12–14, 22, 26]. One remaining publication did not specify patient age, although they can be presumed to be adult based on contextual information about the sample [16].

**Fig. 3:**
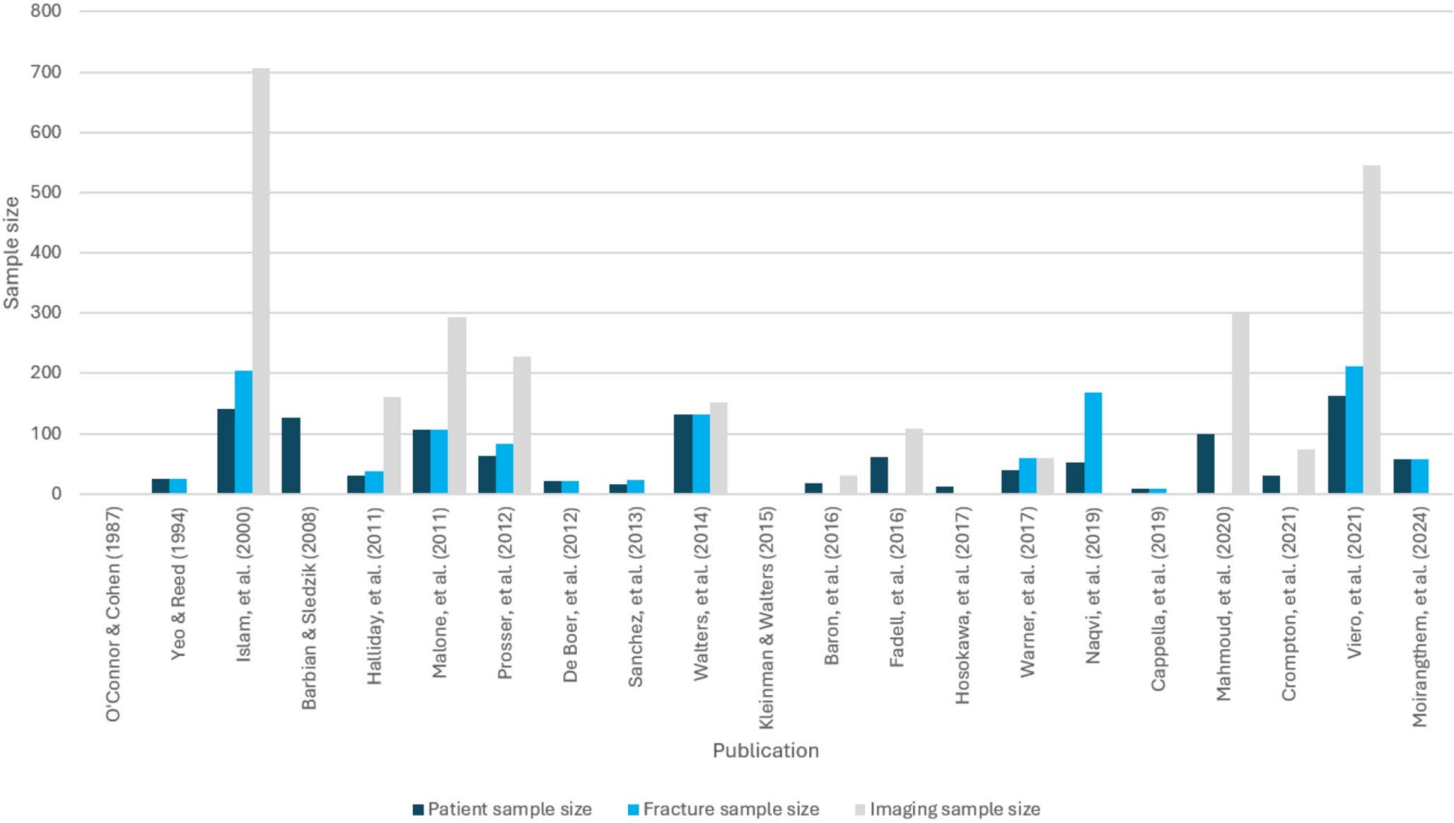
Bar chart showing the number of patients, fractures, and images (e.g., radiographs, MRI scans, and histology slides) assessed in each publication

19 of the 21 publications specified the number of patients from which the ageing methods were developed [6, 12–29] (Fig. 3). The remaining two [11, 30] were book chapters from two different editions of Kleinman’s ‘Diagnostic Imaging of Child Abuse’ where the timetables of healing were developed from the author’s clinical experience, meaning no fracture sample was described. For the 19 publications in which patient number was specified, this ranged from 8 to 162, with a mean of 63.21 patients per study. Similarly, 13 papers specified the number of fractures assessed [6, 14, 16, 18, 20, 22–29], which ranged from 9 to 212 (mean 87.54). Lastly, 11 publications detailed the number of images reviewed in each study, such as the number of individual radiographs or histology slides [6, 13, 15, 17, 18, 20, 21, 24, 26–28]. This ranged from 31 to 707 (mean 241.91).

**Fig. 4:**
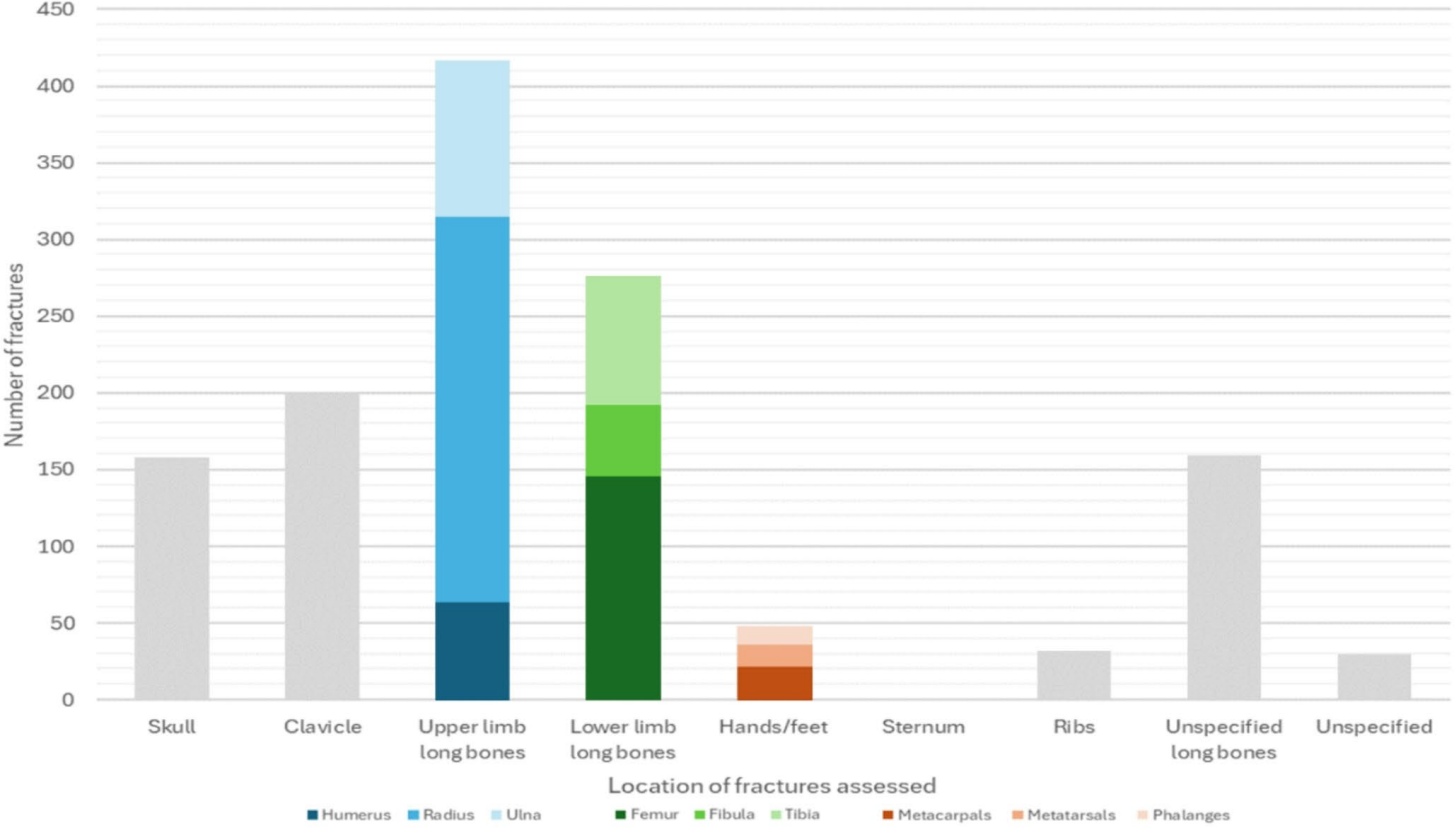
Bar chart showing the number of samples per anatomical region studied across all publications

Finally, reviewing the region of the body studied (Fig. 4), most samples (*n* = 851/1289, 66.02%) were from long bones, especially the radius (*n* = 250, 19.39%). The frequency and percentage data reported here reflects 20 of the 21 papers in full. For the remaining paper [22], it was stated that 30 samples were combined from the clavicle, humerus, ulna, ribs, pelvis, patella, and tibia, but the number for each bone was not given (these form the ‘Unspecified’ column in Fig. 4). For the skull, 158 (12.26%) samples were assessed, meanwhile 32 (2.48%) samples were of the ribs.

Of the 19 papers specifying the bone fracture sample they studied, the exact age of the fracture was known in 14 of these, for example being a birth related injury, or through detailed clinical notes [6, 12–15, 17–19, 21, 23, 24, 26–28]. For a further three papers [20, 22, 29], it can be assumed that fracture ages were known based on descriptions of the study sample, but this is not definitively stated. De Boer and colleagues’ [16] study using archaeological samples did not specify whether the timing of fractures was known based on historical records. Lastly, Sanchez, et al. [25] did not know the original timing of injuries due to their focus on non-accidental rib fractures, for which clinical histories may have been incomplete or deliberately concealed.

### Imaging modalities

**Fig. 5:**
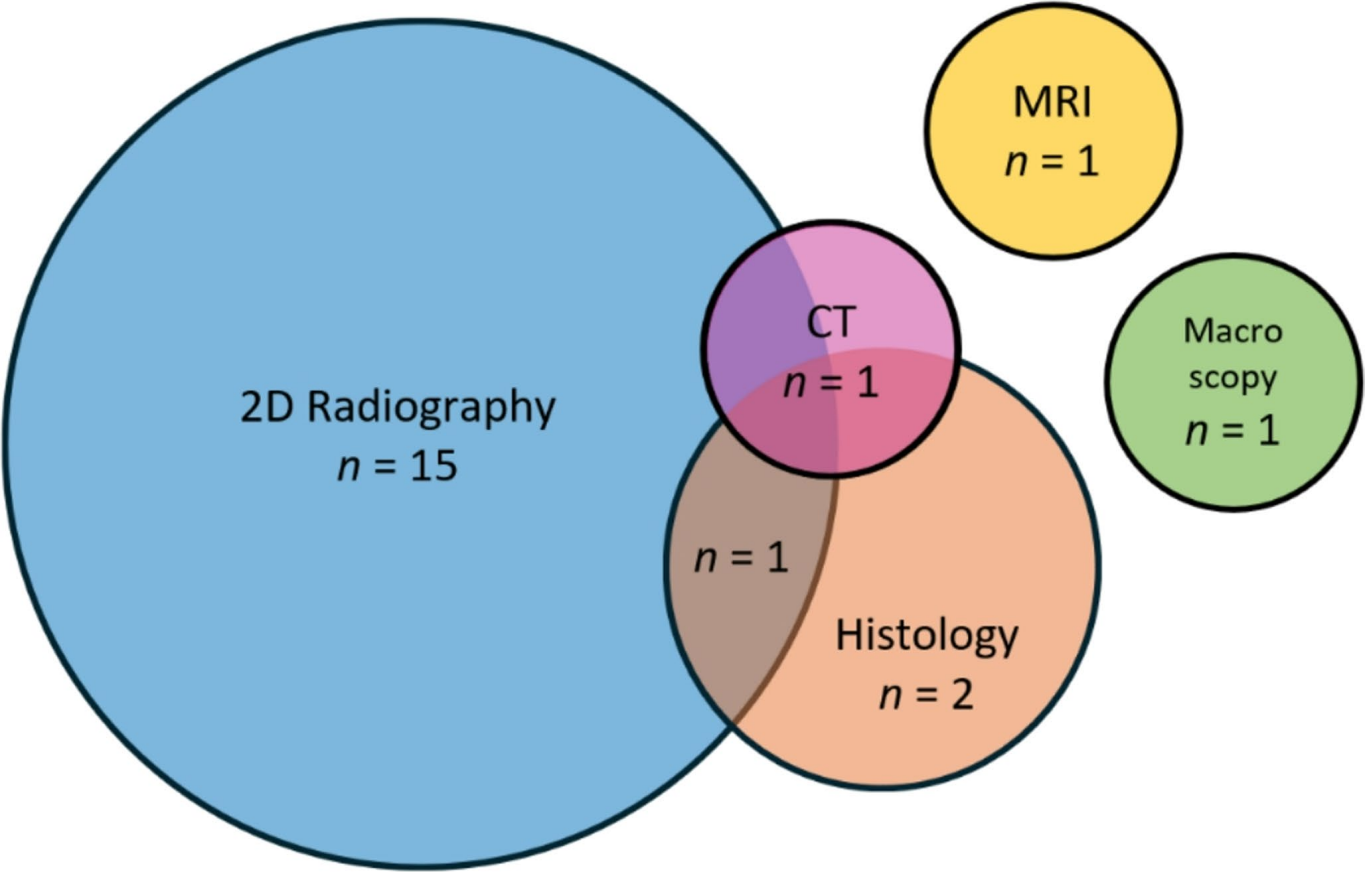
Chart illustrating the distribution of the 21 papers across imaging modality

Of the modalities used to assess fracture healing for ageing, traditional radiographic technologies were by far the most common, with 15 (71.43%) of the 21 published methods using only conventional 2D X-rays [6, 11, 15, 17–21, 24–30] (Fig. 5). One paper [16] compared 2D X-rays with histology, and a further paper [14] combined 2D X-rays with both histology and CT. Two papers used just histology [22, 23], and one paper applied only macroscopic methods to dry archaeological bone samples, with no imaging technology implemented beyond the sporadic use of a light microscope to verify macroscopic observations [12]. Lastly, one paper focused only on MRI [13].

To compare the single most used modality, a summary of all 17 publications using 2D projection radiography, and their associated fracture ageing stages, can be seen in Table 1. The most frequently reported features of healing have been identified and grouped into 7 overlapping and continuous stages, some containing further substages. For practicality, the remaining four modalities using macroscopy, MRI, and histology alone could not be included in the table due to their use of a different suite of healing features than the radiographic methods. In Table 1, to denote time, duration has been specified with d = days, w = weeks, m = months, and y = years. Names are marked with asterisks in the author column when the stages of healing defined in an earlier paper in this systematic review have been incorporated verbatim into the method of a later publication which is also included in this review (e.g., Cappella and colleagues’ [14] (2019) use of the healing stages previously defined by De Boer, et al. [16] in 2012). Under Timing, R is used to denote when timeframes were given as a range from the earliest to latest reported manifestation of the feature. P is the reported peak timing when a feature is most often observed, and M is used where the mean timing of a feature’s appearance was given. Where ‘All long bones’ is given under Fracture location, this means that the sample consisted of at least one of each long bone: humerus, radius, ulna, femur, tibia, and fibula. SPNBF refers to subperiosteal new bone formation. Due to the breadth of variation in the number and nature of healing features assessed across the 17 papers, the data presented in Table 1 represents a summary of the published timetables. This means that in some cases, the terminology titling each stage varies from the wording used in the original method. Additionally, to prevent Table 1 from becoming too large, features that were only studied in a single publication are omitted. Lastly, Hosokawa, et al. [19] studied a sample of 12 infants, seven of whom had no underling disease, while three had osteogenesis imperfecta, one had myotonic dystrophy, and one had arthrogryposis. The time until healing for each group was reported independently, so for consistency with all other publications (of which none studied patients with a known underlying condition), only the times reported for healthy children are included in Table 1.

**Table 1:** Summary of the literature on fracture ageing as visualised with 2D projection radiography

Author	Year	*N* patients	*N* fracture	Fracture location	Patient age	*N* stages/ features assessed	Timing (Range, Peak, Mean)	Radiographically visible stages of fracture healing										
								Stage 1			Stage 2	Stage 3	Stage 4		Stage 5		Stage 6	Stage 7
								No skeletal healing			Fracture gap widening/ granulation	SPNBF/ periosteal reaction	Callus formation		Reduction of fracture line		Bridging	Remodelling
								1a. No healing	1b. Soft tissue swelling	1c. Resolution of soft tissues			4a. Early/ soft callus	4b. Late/ hard callus	5a. Loss of fracture line definition	5b. Sclerosis		
**O’Connor & Cohen**	1987	N/A	N/A	N/A (Clinical experience)	Paediatric	6	R			2 – 21 d		4 – 21 d	10 – 21 d	14 – 90 d	10 – 21 d			3 m – 2 y
							P			4 – 10 d		10 – 14 d	14 – 21 d	21 – 42 d	14 – 21 d			1 y
**Yeo & Reed**	1994	25	25	Femur	0 – 14 y	3	R						9 – 14 d	45 – 66 d			15 – 23 d	
							M						11.7 d	55.6 d			17.2 d	
**Islam, et al.**	2000	141	205	Radius, Ulna	1 – 17 y	8	R				3 – 7 w	2 – 14 w	2 – 14 w	5 – 14 w		3 – 11 w	5 – 14 w	4 – 14 w
							P				4 – 6 w	4 – 7 w	4 – 7 w	13 w		4 – 6 w	13 w	9 w
**Halliday, et al.**	2011	31	37	All long bones	14 d – 44 m	6	R			1 – 10 d		4 d	8+ d	20 – 106 d	12 – 77 d			
**Malone, et al.**	2011	107	107	Radius, Tibia	0 – 5 y	6	R	0 – 14 d			4 – 50 d		4 – 50 d	15 – 75 d	24 – 156 d		24 – 93 d	42 – 750 d
							M	3.3 d			21 d		21 d	38.4 d	65.2 d		43.9 d	313.3 d
Prosser, et al.*	2012	63	82	All long bones, Clavicle	0 – 5 y	6	R		1 – 31 d			5 – 96 d	12 – 66 d	19 – 96 d			19 – 300 d	45 – 421 d
							P		1 – 2 d			15 – 35 d	22 – 35 d	≥ 22 d			≥ 36 d	≥ 36 d
De Boer, et al.**	2012	21	22	All long bones, Sternum, Ribs, Metacarpals	Not reported	6	R						10 – 12+ d	15 – 17+ d			21 – 28+ d	2 – 3+ m
**Sanchez, et al.**	2013	16	23	Ribs	1 – 11 m	6	R	0 – 1 w				1 – 3 w	3 – 5 w	5 – 7 w		9 – 11 w		7 – 9 w
Walters, et al.***	2014	131	131	Clavicle	0 – 3 m	2	R	0 – 7 d				8 – 10+ d	9 – 15+ d	9 – 15+ d				
**Kleinman & Walters**	2015	N/A	N/A	N/A (Clinical experience)	Paediatric	4	R					6 – 21 d	9 – 21 d	14 – 90 d	10 – 21 d			
							P					10 – 14 d	15 – 21 d	21 – 42 d	14 – 21 d			
Fadell, et al. (using *)	2016	61	Not specified	Clavicle	0 – 2 m	4	R					7 – 49 d	11 – 61 d	11 – 61 d			20 – 63 d	35 – 151 d
							1^st^ P					11 d	12 d	12 d			22 d	49 d
							2^nd^ P					42 d	61 d	61 d			63 d	59 d
Warner, et al. (using *)	2017	40	59	Humerus, Femur	9 d – 12 m	4	R					7 – 130 d	9 – 130 d	9 – 130 d			15 – 130 d	51 – 247 d
							P					9 – 49 d	9 – 26 d	9 – 26 d			15 – 67 d	51 – 247 d
**Hosokawa et al.**	2017	12	Not specified	Femur	Newborn (healthy)	3	R					9 – 23 d	10 – 23 d	16 – 32 d				
Cappella, et al. (using **)	2019	8	9	Skull, Ribs	34 – 84 y	6	R						10+ d	15 – 17+ d			21+ d	2+ m
Mahmoud et al. (using *)	2020	100	Not specified	Long bones (unspecified)	1 – 18 y	6	R		< 7 d			8 – 35 d	8 – 35 d	≥ 36 d			≥ 36 d	≥ 36 d
Crompton, et al. (using ***)	2021	30	Not specified	Femur	1 – 33 m	3	R					7 – 59 d	15 – 198 d	15 – 198 d				26 – 198 d
**Viero, et al.**	2021	162	212	All long bones, Metacarpals, Hand phalanges, Metatarsals	21 – 97 y	7	R	1 – 82 d			9 – 80 d	15 – 80 d	22 – 179 d	22 – 179 d		23 – 142 d	30 – 160 d	29 – 385 d
							M	11 d			23.5 d	40.9 d	68.6 d	68.6 d		51.4 d	76.3 d	147.7 d

### Review of healing stages and assessment criteria

**Fig. 6:**
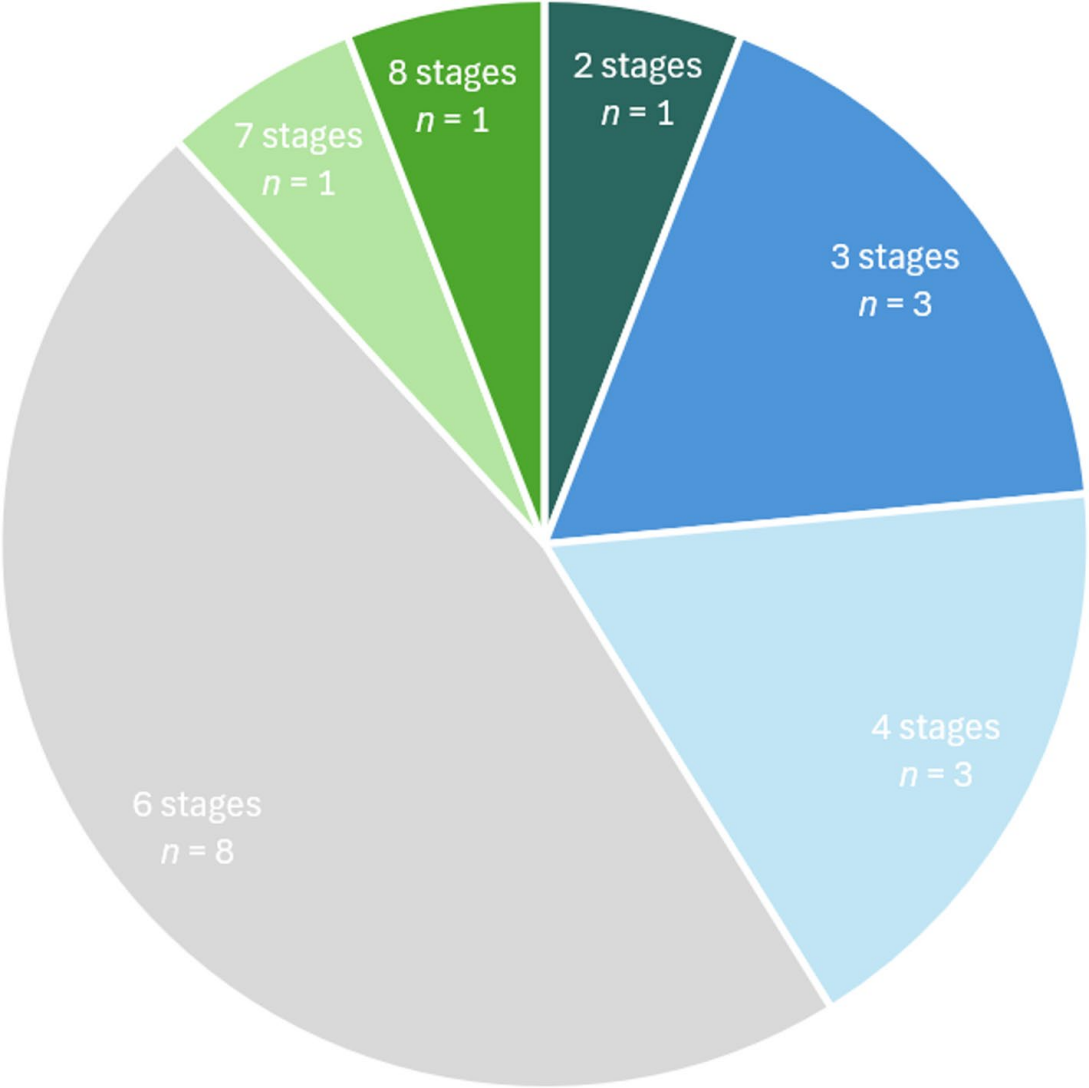
Pie chart showing the number of healing stages assessed to estimate fracture age across the 17 papers using 2D x-rays. *N* numbers represent the number of papers using the specified number of stages

For the 17 papers using 2D X-ray methods (Table 1), the number of stages used to assess healing to estimate fracture age ranged from 2 to 8, with a mean of 5 and a mode of 6. The use of 6 or fewer stages to assess age was more common (Fig. 6), with only 2 timetables [20, 26] detailing more than 6 stages. Whether the timing of each stage was presented as a timetable to guide practitioners, or as more continuous data on when the bony features were observed by a majority of patients, varied by publication. Additionally, some studies focused only on a subprocess of healing, such as Yeo & Reed’s [29] provision of three stages only for the healing callus. Furthermore, just two papers [6, 26] defined a ‘no healing’ stage at the start of the timeline in their method, but a further two [25, 27] reported the period of time before healing was first detectable in the results (Table 1).

Comparing the data in Table 1, it can be seen across all 17 papers, that the only stage consistently reported in all methods was callus formation, with soft and hard callus distinguished as separate stages in 12 papers, and a single callus timeframe given in the remaining 5 papers. Periosteal reaction was then the second most frequently documented stage, described in 13 of the 17 papers. This was followed by remodelling, featured in 12 papers, and bridging which was noted in 10 papers. The remaining stages were infrequently reported, with loss of fracture line definition featured in 4 papers, no healing, fracture gap widening, and sclerosis each in 3 papers, and lastly soft tissue swelling and resolution of the soft tissues, which were both only documented in two papers.

However, the terminology used to name each healing feature (or stage) varied across the papers. To tabulate the data into Table 1, the most frequently used terms for each biological process were used. The ages given in Table 1 are therefore reported in the column for the stages that matches or best fits the process definition given in the original publications.

Considering the histological literature, the number of stages assessed was notably greater. Although Moirangthem, et al. [22] only described 4 stages, these were all situated within the first 5 days after injury. In comparison, De Boer, et al. [16] identified 13 histologically visible features associated with fracture age extending up to 1–2 + years after trauma, while Navqi and colleagues’ [23] give 14 features for infants aged ≤ 1 year.

For the one paper in which CT was used to age fractures, Cappella and colleagues’ [14] implemented the previously defined 13 stages of De Boer, et al. [16], and used CT in combination with 2D X-rays and histology to score a series of histomorphological healing features in a modern adult postmortem sample.

Lastly, one paper used MRI. Baron, et al. [13] identified 9 quantifiable features of healing, spanning 6 stages (or ‘phases’) and 3 substages for ageing.

## Discussion

### Limitations of the fracture demographics studied

Comparison of the literature immediately identifies some limitations in the generalisability of the ageing methods applied due to the bone samples from which the age stages were developed. Specifically, although all 21 papers, acknowledged the importance of accurately ageing injuries for forensic applications, the published methods of fracture ageing are predominantly of a clinical origin, which may not be fully representative of the challenges experienced in a forensic context. For example, many of the clinical radiographs are acquired to assess alignment of the fracture during treatment [31, 32], which may be assisted through use of immobilisation aids such as plaster casts [15]. These negatively affect image quality [15, 18], thereby potentially reducing the level of detail detectable on the radiographs from which to assess healing. Furthermore, victims of non-accidental injuries may have delayed or prevented access to medical treatment, and hence with no immobilisation, secondary microtraumas may occur at the mobile fracture site [24], and timelines of healing developed from immobilised fractures may therefore not be applicable [15, 27, 30].

Additionally, bone samples assessed in the ageing literature do not always reflect the areas of greatest forensic need. Walters, et al. [27] and Fadell, et al. [17], for example, studied birth-related clavicular fractures. This is because clavicular fractures are not immobilised with plaster casts, therefore mitigating issues of reduced image quality due to casting. Additionally, the fractures are of known age due to their occurrence at birth. However, these injuries are rarely ascribed to abuse and are therefore not necessarily representative of the injuries more frequently assessed for forensic purposes [25]. Furthermore, the clavicle undergoes a unique growth process, wherein it is formed through both endochondral and intramembranous ossification [33]. Since long bones develop through endochondral ossification only, this may potentially render healing studies on the clavicle as less generalisable to other bones [17].

The most frequently studied bone was the radius, which made up 19.39% of all samples when pooling the 20 papers. This is likely a reflection of the radius being the most frequently accidentally fractured bone during childhood [34–36], thereby offering a high number of clinical radiographs for research. The fracture locations most relevant for forensic purposes, such as to the rib cage and long bone metaphyses are often underrepresented, despite being among the most suspicious injuries for non-accidental abuse, especially in infants [2, 37, 38]. Indeed, ribs, as evidenced here, are one of the least studied bone locations, being assessed in only 4 publications [14, 16, 22, 25].

### Limitations of the imaging modalities

Beyond the types of bone samples assessed, limitations are further seen throughout the literature due to the often-incomplete data on fracture ageing in the later stages of healing. Antemortem radiographic studies benefit from their ability to provide a more longitudinal insight into healing over time. The use of imaging is generally determined by clinical need with follow-up imaging in the later stages of healing often being minimal [25], as the clinical value does not justify the ionising radiation dose to the patient (e.g. [15, 17]). Healing progression is therefore well documented during the immediate post-injury period, but without regular imaging, it is difficult to ascertain precisely when each stage of healing becomes radiographically visible. This issue becomes increasingly pronounced through the later stages of healing [24].

This issue in data completeness is also observed in histological methods. Bone histology is widely accepted to be the gold standard of fracture ageing, with its superior resolution enabling features of bone healing to be visualised at a cellular level [23, 39, 40]. This is especially beneficial during the first weeks after injury, when fractures progress through a predictable cascade of cellular events that cannot be identified radiographically [14, 16, 23]. In the forensic context, this is especially beneficial as it allows greater precision in estimating the time elapsed between a trauma and death. However, like radiographic methods, the ageing of fractures using histology becomes increasingly variable, imprecise, and broad as time since injury increases (e.g. [16, 23]).

Being destructive, histology is also an exclusively postmortem analysis and can therefore only offer a snapshot into the status of healing at the time of death. This means that for antemortem purposes, a precise and reliable method of fracture ageing is still lacking. This is needed not only for investigations of suspected abuse [24, 27, 28], but also for clinical purposes to monitor the progress of fracture healing with a view to adjust treatment plans if the fracture has not achieved the expected level of healing within a given timeframe [1, 32, 41].

Accordingly, a primary objective of Baron and colleague’s [13] research using MRI was to develop a method of fracture ageing without the limitation of ionising radiation for living individuals. Promisingly, the results found that the early healing phases were characterised by greater definition between T1 and T2 sequences, likely reflecting the greater activity of soft tissues processes like inflammatory response in the initial weeks after injury. These values then decreased overtime in a pattern broadly reflecting the wider radiographic literature where estimates of fracture age become broader with increasing post-injury interval (e.g. [6, 24, 26]). However, in Baron and colleagues’ study [13], MRI was not used to visualise the more subtle features of healing such as loss of fracture line and bridging, which occur between the stages of callus formation and remodelling (Table 1). This may be because MRI is less sensitive than radiography for diagnosing subtle skeletal injuries like rib fractures and metaphyseal lesions [5, 42], although potential soft tissue indicators of fractures such as oedema can be seen [5, 42]. Furthermore, bone features identifiable on MRI may persist for weeks or months after initial appearance, reducing their ability to be precisely aged. As such, MRI may have limited value for ageing fractures beyond the acute timeframe [5].

### Limitations of the ageing methods

Beyond the types of samples and imaging modalities assessed, the timetables developed from them for fracture ageing also face further limitations. Foremost is the lack of terminological standardisation which hinders comparison between methods due to uncertainty that the bony features used to assess healing are being described and diagnosed consistently across studies. This is compounded by differences in the combination of healing features described in each timetable. Although the most common number of stages assessed was six, no two six-point systems assessed the same combination of features. SPNBF, callus formation, and remodelling were most consistently reported, but even this was not unanimous. This continual introduction of new healing stages and definitions has often been justified under the provision that the research is preliminary and requires validation using a larger number of fractures [15, 29, 43]. However, to date just one paper was published with the explicit intent to validate a pre-published method; Cappella and colleague’s [14] test of De Boer, et al. [16]. As such, existing methods of fracture ageing are primarily based on a low volume of data and lack robust scientific validation from secondary research.

Further demonstrating this, Cappella, et al. [14] was the only study in this systematic review to use cross-sectional CT imaging for fracture ageing, which they used in combination with histology. Although they found that CT could offer a more accurate and precise estimate of fracture ages than 2D radiography, histology nonetheless remains the superior modality. They suggest therefore that CT may serve as a complement to histology, but that the precision of CT will remain limited as long as it must use stages of ageing developed from 2D radiography, which are often subjective and vague. Indeed, a fracture in their study was inaccurately aged possibly due to unclear definition of the term ‘union’ in De Boer and colleagues’ [16] original method. They also highlight further subjectivity and inconsistency more generally throughout the stages, which is a concern echoed broadly throughout the literature [5, 8, 44].

The subjective basis to many ageing methods may then account for the broad variability in observer agreement often noted within studies. The most critical conclusions were reached by Halliday, et al. [18], who found SPNBF to be the only one of six healing features reliably identified between observers, despite their high level of professional experience. Describing their results as “disappointing and surprising”, the authors conclude that the radiographic analysis of healing features is unreproducible, and advocate caution in their use for ageing fractures. Throughout the remaining publications reported levels of interobserver agreement more often ranged from moderate to excellent. This reflects variation between studies in the definitions of ageing stages, the combination of features assessed, level of observer experience, or byproducts of the imaging technology such as image quality. For the above reasons, ageing fractures using radiographic methods is therefore considered inexact, and are cautioned when used especially for forensic purposes [5, 7, 18, 45].

### Outside of the systematic review

It is important to acknowledge that this is a systematic review, as opposed to a literature review. As such, while it did not purposefully exclude any imaging modalities or research publications, some were not captured due to the inclusion/exclusion criteria. For example, previous systematic reviews by Prosser, et al. [3] and later updated by Messer, et al. [8] focussed on the radiological dating of paediatric fractures. The former identified just three suitable publications to be included in their review [20, 29, 46], all of which acknowledged the continuous and overlapping nature of fracture healing. Prosser et al. [3] concluded fracture ageing to be an “inexact science”, reporting little consistent data between the three papers, and advocated the adoption of larger-scale studies with the aim to achieve a more consistent and standardised understanding of fracture healing. Later in 2020, Messer and colleagues [8] then reiterated this. Their updated review identified 10 publications [6, 17–20, 24, 25, 27–29], all of which are included in this current systematic review. Their conclusions similarly found variation both between the timelines of fracture healing, as well as research methodologies themselves. They also noted variation in the terminology and definitions used to assess healing radiographically and found that between the different studies there were differences in the ranges, mean and peak times, and first and last appearance dates of different healing features, all of which complicated comparison.

Perhaps part of the challenge in standardisation is that within the limited body of research, many methods draw upon and synthesise the stages of previously published methods. For example, the radiographic ageing stages defined by Hufnagl ([47] not included in this review due to being an unpublished thesis), provided the basis for Malone and colleagues’ [6] timetable, and in turn drew upon the stages outlined by Toal & Mitchell [48] and Hendrix [49] (the former excluded for using an animal sample, and the latter for not providing defined stages of healing). Tritella, et al. [50] similarly proposed the Adapted Fracture Healing Scale (AFHS) based on the earlier work of Malone, et al. [6], Prosser, et al. [24], and de Boer, et al. [16]. This AFHS was then later used by Viero, et al. [26], and is summarised in Table 1 accordingly. When trialled by five observers, the scale was found to perform well, with the highest level of agreement seen between radiographically trained practitioners, i.e., two radiologists and a forensic anthropologist, as compared to the pathologist and orthopaedist. Additionally, it was seen that agreement between observers improved when broader features of healing were assessed. This is demonstrated when categorising callus as simply present or absent, rather than attempting to differentiate between early ‘fluffy’ callus and well-demarcated mature callus [50]. In other words, consistency increased as specificity decreased.

Drury & Cunningham [43], however, provide one of the only direct comparisons of ageing estimates between three different methods [6, 20, 24]. Rather than attempt to derive a new method by synthesising pre-published works, the authors assessed the agreement of each method by using each to estimate the age of 112 long bone fractures across 96 children, where actual fracture age was unknown. They found that while none of the methods were in perfect agreement, the highest level of similarity was seen between Islam, et al. [20] and Malone, et al. [6]. Nonetheless, differences in results still included variation in the duration of the time range estimated, as well as the start and end dates of the range, with some estimates differing by almost 2 months. Although this highlights concern over variation in the time estimates achieved by each method, it is an important result that encourages similar research in the future to test the methods on fractures of known age, to better understand the accuracy and consistency between each method.

### Outlook and directions for future research

It is important that future research remains aware of the challenges highlighted within this paper to help manage and potentially address them. Of high priority should be testing the methods on fractures of known age. This should be conducted similarly to Drury and Cunningham’s [43] comparison of agreement between three methods, but with a view to test their accuracy and precision, as well as consistency in the results they generate.

It would be beneficial to test the methods on a larger sample size of fractures- a need acknowledged in several of the papers in this review (e.g. [6, 13, 29]). Ideally this should include collecting data on different patient and fracture demographics to better understand the role of inter and intra-individual variation, such as the effect of fracture location and patient age on healing rate [5, 14, 26],. For example, infants are often reported to have a faster healing rate than older children and adults [3, 6, 7, 51, 52]. As early as 1988, Skak and Jensen [53] investigated the total time taken to achieve healing union in 275 diaphyseal femoral fractures from 265 children. While they reported a log-normal correlation between age of the patient and time required to achieve union, they acknowledge that this fit was only achieved through the exclusion of patients with delayed union and pseudoarthrosis from the teenage population. The authors suggested that this reflected the increasing variation in healing time with advancing age, and indicates a less predictable model of trauma ageing for older individuals [53]. Despite this, many fracture timelines, namely Yeo & Reed [29], Islam, et al. [20], and Mahmoud, et al. [21], have nonetheless been developed using a combined paediatric demographic, ranging from neonates, infants, and children to older teenagers. The combined timelines may therefore be skewed and not accurately representative of the healing rates of each age group [25]. As such, where sample size permits, it is advisable to study younger children separately from older children and adolescents, specifically isolating those aged ≤ 2 years, whom in the context of abuse commonly have fractures [7, 54–56], and therefore form the demographic on whom fracture ageing is most often performed for forensic purposes [28].

Additionally, immobilisation may affect healing rate as the fracture ends are already in contact, meaning that the extent of SPNBF and callus formation is reduced, and mature lamellar bone is deposited earlier [15, 57]. This has not been tested so any effect on ageing is not understood [15]. and it is not clear whether ageing methods developed from immobilised fractures are applicable to non-immobilised injuries. While the role of immobilisation cannot be experimentally tested using a clinical human sample due to the ethical implications, it is nonetheless a factor that researchers should remain mindful of when comparing results across different methods in which different immobilisation techniques may have been used.

While acknowledging that the breadth of biological variation means that estimates of fracture age will never be absolute, the use of a method less dependent on practitioner experience may also help to support its role in medicolegal investigations [7]. In 2021, Hržić, et al. [58] were the first to use artificial intelligence (AI) as a tool to support the process of ageing paediatric fractures. The preliminary study analysed 7140 wrist radiographs spanning 3570 fractures, with the age of each categorised into one of three groups (fractures aged 0 weeks old, 1–3 weeks old, or older than 3 weeks). They concluded that although the model requires further refinement, it showed promisingly accurate results by assigning fractures to the group of known correct age when information regarding the patient’s sex and age were incorporated. One year later, Kyllonen, et al. [59] then tested a similar machine learning approach. A total of 1,813 radiographs spanning 942 paediatric fractures were aged using the Malone, et al. [6] method, as well as a new four-feature scoring criteria describing callus appearance, fracture discontinuity, bridging, and sclerosis. They found that although differences between the two methods were statistically insignificant, manual assessment using Malone, et al. [6] still offered greater precision. Tsai, et al. [60] similarly used a deep learning algorithm to date clavicular fractures which, like Walters, et al. [27] and Fadell, et al. [17], focused on birth-related injuries to ensure fractures were of known age. Although their model accurately estimated fracture age to within 1 week in 83.7% of cases, the sample was limited to infants aged 3 months or less. Given that they still found accuracy to decrease as time since injury increased and radiographic healing features became less distinct, this suggests that the model may not be applicable to non-birth related fractures in older patients and may not succeed the accuracy of age estimations already possible using manual methods.

AI represents a rapidly developing field and has demonstrated some early potential for fracture ageing. However, current constraints in its accuracy are primarily a byproduct of the resolution of the imaging modality, among other variables such as bone sample and patient demographics. With histology remaining the gold-standard for ageing, this suggests that until radiographic image quality is improved to visualise finer features of healing, ageing will remain broad and nonspecific regardless of its manual or automatic assessment.

To help address this, several publications [61–63] are calling for research to investigate the potential of other imaging modalities for fracture ageing. Accordingly, micro-CT is now among the most frequently cited modalities presenting a promising direction for future research [31, 62–65]. While medical CT scanners can generally offer a resolution down to 600 μm (or 0.6 mm) [66, 67], micro-CT has shown the capability to scan forensic samples for fracture detection in child abuse cases with a resolution as low as 60 μm (or 0.06 mm) [68], where higher resolution can be achieved with smaller samples [68, 69]. Depending on sample size, this gives micro-CT the unique ability to visualise and measure 3D structures with resolution potentially comparable to histopathology [70], and has seen an increasing uptake in forensic practice over recent years [39, 62, 64, 68, 71]. It is acknowledged that micro-CT is an exclusively postmortem analysis, thereby limiting its clinical applications. This is mainly due to the high radiation doses that may cause adverse biological effects, as well as limitations in sample sizes and accessibility to the equipment [72, 73]. Additionally, there are challenges associated with slow data acquisition and processing times, and storage of the large volumes of data generated [73]. Nonetheless, recognising its potential as a postmortem analysis, micro-CT has become the focus of extensive experimental research on fracture ageing. Morgan and colleagues [74], for example, used of a murine model to assess changes in internal callus microstructure overtime, as correlated with the regain of mechanical function. Although their research was clinically aligned, the results demonstrated the potential of micro-CT for fracture ageing, with changes in tissue mineral density, as measured though grayvalue, exhibiting a strong temporal correlation.

More recently, Steyn and colleagues’ [75] then assessed posttraumatic survival time in cranial trauma. Like Cappella, et al. [14], Steyn, et al. [75] used the histomorphological stages of De Boer, et al. [16] to assess healing in dry bone samples, however instead supplemented the histological and radiographic methods with micro-CT [75]. The results demonstrated that although histopathology still offered the highest resolution, micro-CT could valuably visualise some healing features such as early callus formation that were otherwise unable to be detected radiographically. Tsai, et al. [76] and later Baier, et al. [39, 62] then further demonstrate the utility of micro-CT as a 3D complement to histology for fracture detection, finding equally promising results. Micro-CT is therefore increasingly emerging as a technology used in forensic casework to assist in the detection and ageing of fractures, and improve the lucidity of evidentiary presentation in court [39, 62, 68].

Considering micro-CT’s potential as a standalone method for fracture age estimation, initial work by Viero and colleagues [63] has additionally sought to compare micro-CT with 2D projection radiography for the assessment of fracture healing. Using six defined healing stages, they qualitatively assessed the morphology of nine rib fractures of known age, comparing the extent of healing information attainable with both micro-CT and 2D projection radiography. Unsurprisingly, the former offered significantly higher detail for visualising internal callus microstructure and was concluded to be a promising modality for fracture ageing. Given that the study was still reliant on the practitioner’s subjective interpretation of morphological features, Viero, et al. [31] then went on to explore the potential for a more quantitative measure of fracture age. Using the same fracture sample as their previous publication [63], the authors identified five histomorphometric parameters measurable in the healing callus; namely anisotropy, connectivity density, mineralisation volume, and trabecular separation and thickness. It was found that these consistently demonstrated measurable differences associated with age and maturity of the callus, and after further validation may offer a more quantitative and objective means to estimate fracture age. This would then help to further build upon the earlier findings of Sanchez, et al. [25], who found that even when using 2D projection radiography, measurement of callus thickness was a more reliable estimate of fracture age than the qualitative criteria traditionally used. Further research is now needed to expand upon the work of Viero, et al. [31] using a larger sample of fractures with more regular post-traumatic intervals to gain a more precise understanding of the quantifiable evolution of fracture healing and associate this with a usable timetable. The high resolution, non-destructive, and 3D nature of micro-CT therefore situates it as an ideal modality through which to complement plain 2D bone histology and advance novel, more robust methods for objective fracture age estimation.

## Conclusion

It is clear that the estimation of fracture age is a multidisciplinary endeavour, with both clinical and forensic applications. Comparison of the numerous modalities and healing criteria used to age fractures has revealed little methodological consistency between or within modalities, and few attempts to validate approaches for forensic application. While histopathology offers the greatest sensitivity for fracture analysis, it is only applicable to postmortem samples, meaning that radiographic approaches represent most of the published methods. Despite this, low resolution, small sample sizes, inconsistent bone type and patient samples, and the subjective and variable definition of age stages are still among the factors that limit current radiographic applications. Accordingly, the more recent utilisation of industrial imaging modalities for fracture ageing such as micro-CT has seen promising results, however this is yet to be widely researched. Further work is therefore needed to synthesise the strengths of the existing approaches with modern advancements in imaging technology, to advance more holistic and sensitive approaches for fracture ageing.

## References

[1] Roth TD, Ladd LM, Kempton LB (2017) Fracture healing and imaging evaluation. Curr Radiol Rep. 10.1007/s40134-017-0221-0

[2] Marine MB, Forbes-Amrhein MM (2021) Fractures of child abuse. Pediatr Radiol 51:1003–1013. 10.1007/s00247-020-04945-1 33783574 10.1007/s00247-020-04945-1

[3] Prosser I, Maguire S, Harrison SK, Mann M, Sibert JR, Kemp AM (2005) How old is this fracture? Radiologic dating of fractures in children: a systematic review. Am J Roentgenol 184:1282–1286. 10.2214/ajr.184.4.01841282 15788611 10.2214/ajr.184.4.01841282

[4] Crown Prosecution Service (2024) Homicide: murder, manslaughter, infanticide and causing or allowing the death or serious injury of a child or vulnerable adult. Crown Prosecution Service. https://www.cps.gov.uk/legal-guidance/homicide-murder-manslaughter-infanticide-and-causing-or-allowing-death-or-serious. Accessed 31 March 2025

[5] Pickett TA (2015) The challenges of accurately estimating time of long bone injury in children. J Forensic Leg Med 33:105–110. 10.1016/j.jflm.2015.04.012 26048508 10.1016/j.jflm.2015.04.012

[6] Malone CA, Sauer NJ, Fenton TW (2011) A radiographic assessment of pediatric fracture healing and time since injury. J Forensic Sci 56:1123–1130. 10.1111/j.1556-4029.2011.01820.x 21644992 10.1111/j.1556-4029.2011.01820.x

[7] Paddock M, Choudhary AK, Jeanes A, Mankad K, Mannes I, Raissaki M, Adamsbaum C, Argyropoulou MI, van Rijn RR, Offiah AC (2023) Controversial aspects of imaging in child abuse: a second roundtable discussion from the ESPR child abuse taskforce. Pediatr Radiol 53:739–751. 10.1007/s00247-023-05618-5 36879046 10.1007/s00247-023-05618-5 PMC10027646

[8] Messer DL, Adler BH, Brink FW, Xiang H, Agnew AM (2020) Radiographic timelines for pediatric fracture healing: a systematic review. Pediatr Radiol 50:1041–1048. 10.1007/s00247-020-04648-7 32157365 10.1007/s00247-020-04648-7

[9] Harwood PJ, Newman JB, Michael ALR (2010) An update on fracture healing and non-union. Orthop Trauma 24:9–23. 10.1016/j.mporth.2009.12.004

[10] Rethlefsen ML, Kirtley S, Waffenschmidt S, Ayala AP, Moher D, Page MJ, Koffel JB, Group PRISMA-S (2021) PRISMA-S: an extension to the PRISMA statement for reporting literature searches in systematic reviews. Syst Rev 10:1–19. 10.1186/s13643-020-01542-z 33499930 10.1186/s13643-020-01542-z PMC7839230

[11] O’Connor JF, Cohen JC (1987) Dating fractures. In: Kleinman PK (ed) Diagnostic imaging of child abuse. Williams & Wilkins, Baltimore, pp 103–113

[12] Barbian LT, Sledzik PS (2008) Healing following cranial trauma. J Forensic Sci 53:263–268. 10.1111/j.1556-4029.2007.00651.x 18298494 10.1111/j.1556-4029.2007.00651.x

[13] Baron K, Neumayer B, Widek T, Schick F, Scheicher S, Hassler E, Scheurer E (2016) Quantitative MR imaging in fracture dating – initial results. Forensic Sci Int 261:61–69. 10.1016/j.forsciint.2016.01.020 26890805 10.1016/j.forsciint.2016.01.020

[14] Cappella A, de Boer HH, Cammilli P, de Angelis D, Messina C, Sconfienza LM, Sardanelli F, Sforza C, Cattaneo C (2019) Histologic and radiological analysis on bone fractures: estimation of posttraumatic survival time in skeletal trauma. Forensic Sci Int 302:109909. 10.1016/j.forsciint.2019.109909 31404812 10.1016/j.forsciint.2019.109909

[15] Crompton S, Messina F, Klafkowski G, Hall C, Offiah AC (2021) Validating scoring systems for fracture healing in infants and young children: pilot study. Pediatr Radiol 51:1682–1689. 10.1007/s00247-021-05038-3 33847785 10.1007/s00247-021-05038-3 PMC8363550

[16] de Boer HH, van der Merwe AE, Hammer S, Steyn M, Maat GJR (2012) Assessing post-traumatic time interval in human dry bone. Int J Osteoarchaeol 25:98–109. 10.1002/oa.2267

[17] Fadell M, Miller A, Trefan L, Weinman J, Stewart J, Hayes K, Maguire S (2017) Radiological features of healing in newborn clavicular fractures. Eur Radiol 27:2180–2187. 10.1007/s00330-016-4569-y 27629420 10.1007/s00330-016-4569-y

[18] Halliday KE, Broderick NJ, Somers JM, Hawkes R (2011) Dating fractures in infants. Clin Radiol 66:1049–1054. 10.1016/j.crad.2011.06.001 21763645 10.1016/j.crad.2011.06.001

[19] Hosokawa T, Yamada Y, Sato Y, Tanami Y, Oguma E (2017) Subperiosteal new bone and callus formations in neonates with femoral shaft fracture at birth. Emerg Radiol 24:143–148. 10.1007/s10140-016-1462-6 27830345 10.1007/s10140-016-1462-6

[20] Islam O, Soboleski D, Symons S, Davidson LK, Ashworth MA, Babyn P (2000) Development and duration of radiographic signs of bone healing in children. Am J Roentgenol 175:75–78. 10.2214/ajr.175.1.1750075 10882250 10.2214/ajr.175.1.1750075

[21] Mahmoud AS, Fahmy AM, Mahmoud AM, Bayomi AA, El-Amir AY (2020) Dating of long bone fracture healing among Egyptian pediatrics by radiology (x-ray). Indian J Forensic Med Toxicol 14:1048–1052. 10.37506/IJFMT.V14I3.10511

[22] Moirangthem S, Arora A, Vidua RK, Goel G (2024) Autopsy-based comparative study of gross and histopathological findings at bone fracture surfaces before and after death. Am J Forensic Med Pathol 45:111–117. 10.1097/PAF.0000000000000914 38261541 10.1097/PAF.0000000000000914

[23] Naqvi A, Raynor E, Freemont AJ (2019) Histological ageing of fractures in infants: a practical algorithm for assessing infants suspected of accidental or non-accidental injury. Histopathology 75:74–80. 10.1111/his.13850 30820979 10.1111/his.13850 PMC6618162

[24] Prosser I, Lawson Z, Evans A, Harrison S, Morris S, Maguire S, Kemp AM (2012) A timetable for the radiologic features of fracture healing in young children. Am J Roentgenol 198:1014–1020. 10.2214/AJR.11.6734 22528890 10.2214/AJR.11.6734

[25] Sanchez TR, Nguyen H, Palacios W, Doherty M, Coulter K (2013) Retrospective evaluation and dating of non-accidental rib fractures in infants. Clin Radiol 68:e467–e471. 10.1016/j.crad.2013.03.017 23622800 10.1016/j.crad.2013.03.017

[26] Viero A, Obertová Z, Cappella A, Messina C, Sconfienza LM, Sardanelli F, Tritella S, Montisci M, Gregori D, Tagliaro F, Cattaneo C (2021) The problem of dating fractures: a retrospective observational study of radiologic features of fracture healing in adults. Forensic Sci Int 329:111058. 10.1016/j.forsciint.2021.111058 34710653 10.1016/j.forsciint.2021.111058

[27] Walters MM, Forbes PW, Buonomo C, Kleinman PK (2014) Healing patterns of clavicular birth injuries as a guide to fracture dating in cases of possible infant abuse. Pediatr Radiol 44:1224–1229. 10.1007/s00247-014-2995-z 24777389 10.1007/s00247-014-2995-z

[28] Warner C, Maguire S, Trefan L, Miller A, Weinman J, Fadell M (2017) A study of radiological features of healing in long bone fractures among infants less than a year. Skeletal Radiol 46:333–341. 10.1007/s00256-016-2563-8 28070625 10.1007/s00256-016-2563-8

[29] Yeo LI, Reed MH (1994) Staging of healing of femoral fractures in children. Can Assoc Radiol J 45:16–19 8118709

[30] Kleinman PK, Walters MM (2015) Dating fractures. In: Kleinman PK (ed) Diagnostic imaging of child abuse, 3rd edn. Cambridge Univ, Cambridge, pp 208–216

[31] Viero A, Biehler-Gomez L, Messina C, Cappella A, Giannoukos K, Viel G, Tagliaro F, Cattaneo C (2022) Utility of micro-CT for dating post-cranial fractures of known post-traumatic ages through 3D measurements of the trabecular inner morphology. Sci Rep 12:10543. 10.1038/s41598-022-14530-1 35732857 10.1038/s41598-022-14530-1 PMC9218115

[32] Whelan D, Bhandari M, Stephen D, Kreder H, McKee M, Zdero R, Schemitsch EH (2010) Development of the radiographic union score for tibial fractures for the assessment of tibial fracture healing after intramedullary fixation. J Trauma 68:629–632. 10.1097/TA.0b013e3181a7c16d 19996801 10.1097/TA.0b013e3181a7c16d

[33] Ferguson D, Scott B (2016) The enigmatic role and development of the clavicle. Orthop Trauma 30:273–276. 10.1016/j.mporth.2015.05.002

[34] Aygun U (2020) The feature assessment of the bone fractures in 1020 children and review of the literature. North Clin Istanb 7:460–466. 10.14744/nci.2020.82713 33163881 10.14744/nci.2020.82713 PMC7603841

[35] Mustafa Q, Wilson C, Crook T (2025) Distal radius fractures, current evidence including DRAFFT trial. Surg (Oxf) 43:96–101. 10.1016/j.mpsur.2024.12.001

[36] Randsborg P-H, Gulbrandsen P, Benth JS, Sivertsen EA, Hammer O-L, Fuglesang HFS, Arøen A (2013) Fractures in children: epidemiology and activity-specific fracture rates. J Bone Joint Surg Am 95:e42. 10.2106/JBJS.L.00369 23553305 10.2106/JBJS.L.00369

[37] Prosser IM, Harrison SK (2017) Interpreting fractures in child maltreatment. Paediatr Child Health 27:28–32. 10.1016/j.paed.2016.10.003

[38] Raynor E, Konala P, Freemont A (2018) The detection of significant fractures in suspected infant abuse. J Forensic Leg Med 60:9–14. 10.1016/j.jflm.2018.09.002 30196192 10.1016/j.jflm.2018.09.002

[39] Baier W, Mangham C, Warnett JM, Payne M, Painter M, Williams MA (2019) Using histology to evaluate micro-CT findings of trauma in three post-mortem samples – first steps towards method validation. Forensic Sci Int 297:27–34. 10.1016/j.forsciint.2019.01.027 30769301 10.1016/j.forsciint.2019.01.027

[40] Aksoy U, Özkayalar H, Orhan K (2020) Micro-CT in comparison with histology in the qualitative assessment of bone and pathologies. In: Orhan K (ed) Micro-computed tomography (micro-CT) in medicine and engineering. Springer Nature, Cham, pp 109–124

[41] Nicholson JA, Yapp LZ, Keating JF, Simpson AHRW (2021) Monitoring of fracture healing. Update on current and future imaging modalities to predict union. Injury 5:S29–S34. 10.1016/j.injury.2020.08.016 10.1016/j.injury.2020.08.016 32826052

[42] Ross S, Ebner L, Flach P, Brodhage R, Bolliger SA, Christe A, Thali MJ (2012) Postmortem whole-body MRI in traumatic causes of death. Am J Roentgenol 199:1186–1192. 10.2214/AJR.12.8767 23169707 10.2214/AJR.12.8767

[43] Drury A, Cunningham C (2018) Determining when a fracture occurred: does the method matter? Analysis of the similarity of three different methods for estimating time since fracture of juvenile long bones. J Forensic Leg Med 53:97–105. 10.1016/j.jflm.2017.11.004 29227827 10.1016/j.jflm.2017.11.004

[44] Fischer JS, Kazam JJ, Fufa D, Bartolotta RJ (2019) Radiologic evaluation of fracture healing. Skeletal Radiol 48:349–361. 10.1007/s00256-018-3051-0 30238139 10.1007/s00256-018-3051-0

[45] Offiah AC, Hall CM (2009) Radiological atlas of child abuse. CRC, Boca Raton

[46] Cumming WA (1979) Neonatal skeletal fractures. Birth trauma or child abuse? J Can Assoc Radiol 30:30–33 429433

[47] Hufnagl KB (2005) An investigation of time since injury: a radiographic study of fracture healing. Dissertation, Louisiana State University

[48] Toal RL, Mitchell SK (2002) Fracture healing and complications. In: Thrall DE (ed) Textbook of veterinary diagnostic radiology, 4th edn. WB Saunders Company, Pennsylvania, pp 161–178

[49] Hendrix RW (2002) Fracture healing. In: Rogers LF (ed) Radiology of skeletal trauma, 3rd edn. Churchill Livingstone, Philadelphia, pp 203–230

[50] Tritella S, Obertová Z, Sconfienza LM, Collini F, Cristini E, Amadasi A, Ciprandi B, Spairani R, Albano D, Viero A, Cappella A, Cammilli P, Sardanelli F, Cattaneo C (2020) Multi-rater agreement using the adapted fracture healing scale (AFHS) for the assessment of tubular bones on conventional radiographs: preliminary study. J Forensic Sci 65:2112–2116. 10.1111/1556-4029.14541 32809218 10.1111/1556-4029.14541

[51] Rao AG, Hill JG (2014) Non-central nervous system imaging of pediatric inflicted injury. In: Collins KA, Byard RW (eds) Forensic pathology of infancy and childhood. Springer, New York, pp 539–584

[52] Fleischman JM, Soto Martinez ME, Wiersema JM, Pinto DC (2020) The role of the forensic anthropologist in the pediatric autopsy: interpretations, contributions, and challenges. WIREs Forensic Sci. 10.1002/wfs2.1389

[53] Skak SV, Jensen TT (1988) Femoral shaft fracture in 265 children: log-normal correlation with age of speed of healing. Acta Orthop Scand 59:704–707. 10.3109/17453678809149430 3213461 10.3109/17453678809149430

[54] Royal College of Paediatrics and Child Health (2020) Child Protection Evidence – Systematic review on Fractures. Royal College of Paediatrics and Child Health. https://childprotection.rcpch.ac.uk/child-protection-evidence/fractures-systematic-review/ Accessed 3 March 2025

[55] The Society and College of Radiographers and The Royal College of Radiologists (2018) The Radiological Investigation of Suspected Physical Abuse in Children. The Society of Radiographers. https://www.sor.org/learning-advice/professional-body-guidance-and-publications/documents-and-publications/archive-documents/the-radiological-investigation-of-physical-abuse-i#:~:text=It%20revamps%20initial%20guidance%20from%202008%20and%20incorporates,This%20document%20is%20available%20as%20a%20PDF%20only. Accessed 3 March 2025

[56] Colleran GC, Fossmark M, Rosendahl K, Argyropoulou M, Mankad K, Offiah AC (2025) ESR essentials: imaging of suspected child abuse – practice recommendations by the European society of paediatric radiology. Eur Radiol 35:1868–1880. 10.1007/s00330-024-11052-4 39289300 10.1007/s00330-024-11052-4 PMC11914366

[57] Boyd D (2018) The anatomical basis for fracture repair: recognition of the healing continuum and its forensic applications to investigations of pediatric and elderly abuse. In: Boyd CC Jr., Boyd D (eds) Forensic anthropology: theoretical framework and scientific basis. Wiley, Chichester, pp 149–200

[58] Hržić F, Janisch M, Štajduhar I, Lerga J, Sorantin E, Tschauner S (2021) Modeling uncertainty in fracture age estimation from pediatric wrist radiographs. Mathematics 9:3227. 10.3390/math9243227

[59] Kyllonen KM, Monson KL, Smith MA (2022) Postmortem and antemortem forensic assessment of pediatric fracture healing from radiographs and machine learning classification. Biology 11:749. 10.3390/biology11050749 35625477 10.3390/biology11050749 PMC9138832

[60] Tsai A, Grant PE, Warfield SK, Ou Y, Kleinman PK (2022) Deep learning of birth-related infant clavicle fractures: a potential virtual consultant for fracture dating. Pediatr Radiol 52:2206–2214. 10.1007/s00247-022-05380-0 35578043 10.1007/s00247-022-05380-0

[61] Arthurs OJ, Hutchinson JC, Sebire NJ (2017) Current issues in postmortem imaging of perinatal and childhood deaths. Forensic Sci Med Pathol 13:58–66. 10.1007/s12024-016-9821-x 28083782 10.1007/s12024-016-9821-x PMC5306347

[62] Baier W, Norman DG, Williams MA (2021) Micro-CT for the examination of paediatric rib injuries: a case series. Forensic Sci Int 325:110789. 10.1016/j.forsciint.2021.110789 34217913 10.1016/j.forsciint.2021.110789

[63] Viero A, Biehler-Gomez L, Cappella A, Messina C, Montisci M, Cattaneo C (2021) The potential of micro-CT for dating post-cranial bone fractures: a macroscopic, radiographic, and microtomography study of fractures of known post-traumatic ages. Int J Legal Med 135:1913–1921. 10.1007/s00414-021-02582-3 33772611 10.1007/s00414-021-02582-3

[64] Franchetti G, Viel G, Fais P, Fichera G, Cecchin D, Cecchetto G, Giraudo C (2022) Forensic applications of micro-computed tomography: a systematic review. Clin Transl Imaging 10:597–610. 10.1007/s40336-022-00510-y

[65] Akbulut N, Çetin S, Bilecenoğlu B, Altan A, Ocak M, Şen E, Atakan C, Orhan K (2021) Evaluation of the detectability of early mandible fracture healing findings in terms of vitality aspect by using micro-CT technology in postmortem interval. Leg Med 52:101914. 10.1016/j.legalmed.2021.101914 10.1016/j.legalmed.2021.101914 34091405

[66] Alsop K, Norman DG, Baier W, Colclough J, Williams MA (2022) Advantages of micro-CT in the case of a complex dismemberment. J Forensic Sci 67:1258–1266. 10.1111/1556-4029.15007 35118663 10.1111/1556-4029.15007 PMC9305105

[67] Shelmerdine SC, Davendralingam N, Langan D, Palm L, Mangham C, Arthurs OJ, CORNRD Study Collaborators (2024) Post-mortem skeletal survey (PMSS) versus post-mortem computed tomography (PMCT) for the detection of corner metaphyseal lesions (CML) in children. Eur Radiol 34:5561–5569. 10.1007/s00330-024-10679-7 38459348 10.1007/s00330-024-10679-7

[68] Primeau C, Norman DG, Baier W, Goia S, Blaik S, Williams MA (2024) Micro-CT in a forensic examination of a fatal child abuse case: a case report. Sci Justice 64:297–304. 10.1016/j.scijus.2024.04.001 38735666 10.1016/j.scijus.2024.04.001

[69] Rutty GN, Brough A, Biggs MJP, Robinson C, Lawes SDA, Hainsworth SV (2013) The role of micro-computed tomography in forensic investigations. Forensic Sci Int 225:60–66. 10.1016/j.forsciint.2012.10.030 23153801 10.1016/j.forsciint.2012.10.030

[70] Edwards H, Shelmerdine SC, Arthurs OJ (2023) Forensic post-mortem CT in children. Clin Radiol 78:839–847. 10.1016/j.crad.2023.06.001 37827594 10.1016/j.crad.2023.06.001

[71] Norman DG, Baier W, Watson DG, Burnett B, Painter M, Williams MA (2018) Micro-CT for saw mark analysis on human bone. Forensic Sci Int 293:91–100. 10.1016/j.forsciint.2018.10.027 30415097 10.1016/j.forsciint.2018.10.027

[72] Miyahara N, Kokubo T, Hara Y, Yamada A, Koike T, Arai Y (2016) Evaluation of X-ray doses and their corresponding biological effects on experimental animals in cone-beam micro-CT scans (R-mCT2). Radiol Phys Technol 9:60–68. 10.1007/s12194-015-0334-1 26441335 10.1007/s12194-015-0334-1 PMC4722077

[73] Ritman E (2011) Current status of developments and applications of micro-CT. Annu Rev Biomed Eng 13:531–552. 10.1146/annurev-bioeng-071910-124717 21756145 10.1146/annurev-bioeng-071910-124717

[74] Morgan EF, Mason ZD, Chien KB, Pfeiffer AJ, Barnes GL, Einhorn TA, Gerstenfeld LC (2009) Micro-computed tomography assessment of fracture healing: relationships among callus structure, composition, and mechanical function. Bone 44:335–344. 10.1016/j.bone.2008.10.039 19013264 10.1016/j.bone.2008.10.039 PMC2669651

[75] Steyn M, de Boer HH, Van der Merwe AE (2014) Cranial trauma and the assessment of posttraumatic survival time. Forensic Sci Int 244:e25–e29. 10.1016/j.forsciint.2014.08.021 25217847 10.1016/j.forsciint.2014.08.021

[76] Tsai A, McDonald AG, Rosenberg AE, Gupta R, Kleinman PK (2014) High-resolution CT with histopathological correlates of the classic metaphyseal lesion of infant abuse. Pediatr Radiol 44:124–140. 10.1007/s00247-013-2813-z 24481795 10.1007/s00247-013-2813-z

